# Greater Improvements in Vaccination Outcomes Among Black Young Adults With Vaccine-Resistant Attitudes in the United States South Following a Digital Health Intervention: Latent Profile Analysis of a Randomized Control Trial

**DOI:** 10.2196/67370

**Published:** 2025-04-16

**Authors:** Noah Mancuso, Jenna Michaels, Erica N Browne, Allysha C Maragh-Bass, Jacob B Stocks, Zachary R Soberano, C Lily Bond, Ibrahim Yigit, Maria Leonora G Comello, Margo Adams Larsen, Kathryn E Muessig, Audrey Pettifor, Lisa B Hightow-Weidman, Henna Budhwani, Marie C D Stoner

**Affiliations:** 1 Women's Global Health Imperative, Research Triangle Institute (RTI) International, 3040 East Cornwallis Road, Research Triangle Park, NC, 27709, United States, 1 9195416000; 2 Department of Epidemiology, Emory University, Atlanta, GA, United States; 3 FHI 360, Durham, NC, United States; 4 Institute on Digital Health and Innovation, College of Nursing, Florida State University (FSU), Tallahassee, FL, United States; 5 Department of Epidemiology, University of North Carolina at Chapel Hill, Chapel Hill, NC, United States; 6 Virtually Better, Inc, Decatur, GA, United States

**Keywords:** COVID-19, mHealth, African American and Black, young adults, vaccination, mobile health

## Abstract

**Background:**

Negative attitudes toward vaccines and suboptimal vaccination rates among African American and Black (Black) Americans have been well documented, due to a history of medical racism and human rights violations in the United States. However, digital health interventions (DHI) have been shown to address racial disparities in several health outcomes, such as cardiovascular disease, HIV, and maternal health. The Tough Talks COVID (TT-C) study was a randomized controlled trial of a DHI designed to empower Black young adults in the United States South to make informed, autonomous decisions about COVID-19 vaccine uptake by addressing structural barriers and misinformation about vaccines.

**Objective:**

Our objective was to identify subgroups of Black young adults with various vaccine attitudes at baseline and determine the subgroups for which the TT-C DHI was most impactful.

**Methods:**

Black young adults aged 18‐29 years in Alabama, Georgia, and North Carolina who were unvaccinated or insufficiently vaccinated against COVID-19 completed three online surveys over three months (N=360). Latent profile analysis was used to identify subgroups based on general vaccine attitudes at baseline, including hesitancy, confidence, knowledge, conspiracy beliefs, and mistrust. Logistic regression was used to examine the associations between latent profiles and vaccine uptake, and linear regression was used to examine changes in vaccine attitudes at three months post-randomization. Modification of the TT-C DHI’s effects was assessed by latent profiles.

**Results:**

Three latent profiles emerged: vaccine-receptive (n=124), vaccine-neutral (n=155), and vaccine-resistant (n=81). Political affiliation, income, social support, and recent flu vaccination differed significantly between the three subgroups (*P*<.05). Vaccine uptake was not significantly different by subgroup, and the TTC-DHI did not have differing effects on uptake across subgroups. However, the DHI had the strongest effect—with statistically significant measures of association (*P*<.05) and interaction *P* values (*P<*.10)—among the baseline vaccine-resistant and vaccine-neutral subgroups compared to the vaccine-receptive subgroups at three months in improving vaccine hesitancy, confidence, and conspiracy beliefs at three months: vaccine-resistant difference: −0.40 (−0.76 to −0.37), 0.39 (0.02 to 0.75), and −0.47 (−0.86 to −0.09); vaccine neutral difference: −0.36 (−0.52 to −0.19), 0.35 (0.18 to 0.51), and −0.24 (−0.44 to −0.03). The DHI had no effects on these outcomes among the vaccine-receptive subgroup.

**Conclusions:**

Our findings revealed subgroups of Black young adults in the United States South with different vaccination attitudes, for which the TT-C intervention had differing effects. Black young adults who are vaccine-resistant or vaccine-neutral may experience larger gains from a digital vaccine intervention. Future work aimed at improving vaccination outcomes could target these populations to maximize resource efficiency and drive the greatest improvements in vaccine outcomes.

## Introduction

The World Health Organization declared the outbreak of novel COVID-19, a global pandemic in early 2020, caused by SARS-CoV-2 [1]. By the end of the year, two COVID-19 vaccines had been issued emergency use authorizations in the United States [2,3]. At the time, nearly three in five Americans reported intentions to be vaccinated for COVID-19; however, African American or Black (Black) Americans reported intentions to vaccinate approximately 30% lower than that of White Americans and 50% lower than that of Asian Americans [4]. Hesitancy toward COVID-19 vaccination was particularly high among Black young adults and residents of the United States South [5,6], which translated into lower uptake of the COVID-19 vaccine and a disproportionately high mortality rate for Black Americans early in the pandemic [7,8].

Negative attitudes toward vaccines and suboptimal vaccination rates among Black Americans have been influenced by a history of medical racism and human rights violations in the United States [9-14]. Continued harmful experiences with the health care system and government motivate hesitancy toward public health programs, such as vaccination, as a self-protective coping mechanism [15]. Studies have consistently shown this hesitancy, with reported lower vaccine uptake among Black communities for the annual flu shot due to distrust of medical institutions and concerns for safety [16-18]. Additionally, disparities in human papillomavirus vaccination have been observed among young Black girls, which is reflected in higher rates of cervical cancer incidence and mortality later in life [19,20]. During the COVID-19 pandemic, these experiences were exacerbated for Black young adults by the spread of misinformation and conspiracy theories online and on social media [21,22].

Digital health interventions (DHIs) have been successful in addressing racial disparities in health by creating culturally tailored messaging, inclusive programming, and more accessible services for disease prevention, ranging from cardiovascular disease to HIV to maternal health [23-27]. DHIs are particularly helpful for young adults, given the high proportion of Americans aged 18‐28 years with smartphones who report using web-based news and social media sites to access information about COVID-19 [28,29]. However, few DHIs to date have been developed specifically for COVID-19 vaccination among Black young adults in the United States South.

The Tough Talks COVID (TT-C) study was a randomized controlled trial of a DHI designed to empower Black young adults in the United States South to make informed, autonomous decisions about COVID-19 vaccine uptake (ClinicalTrials.gov NCT05490329) [30]. The TT-C intervention was cocreated using community-based participatory research methods to address structural barriers and misinformation about vaccines through interactive activities and digital storytelling [31,32]. Assuming heterogeneity among the study population, we aimed to use a latent profile analysis (LPA) to identify subgroups of Black young adults with different baseline vaccine attitudes and identify subgroups for whom TT-C was most impactful, to allow for better-informed community-based policies, practices, and messaging related to vaccine uptake and attitudes.

## Methods

### Participants

We used data from the TT-C randomized control trial, which included Black young adults aged 18‐29 years who were unvaccinated or insufficiently vaccinated against COVID-19 in Alabama, Georgia, and North Carolina. Participants were recruited online or via social media and randomized 1:1 to either the TT-C DHI, a self-directed asynchronous app developed to enable Black young adults in Southern United States to make decisions about COVID-19 vaccine receipt via nonstigmatizing and tailored messaging, or an information standard-of-care control consisting of COVID-19 vaccine materials adapted from the Centers for Disease Control and Prevention for three months. Surveys were completed at baseline, one month, and three months after randomization. Additional details about the study, which was conducted between March and December 2023, have been published previously [30] .

### Latent Indicators

Five baseline variables related to general vaccine attitudes at baseline were used as latent indicators in the LPA: vaccine hesitancy, vaccine confidence, vaccine-related conspiracy beliefs, vaccine knowledge, and group-based medical mistrust. Each variable was assessed using a previously validated scale with a standardized continuous score as input using z-scores, such that the mean score transformed to zero to remove the impact of any baseline differences in means or variances across the variables before they were used in the LPA. This approach helps mitigate the influence of baseline distribution differences in the clustering process by making the variables comparable. The measures used in this LPA were prespecified in the study protocol and tested in formative surveys with Black young adults [30,33]. Vaccine hesitancy was a mean score of a 9-item scale with Likert responses ranging from 1‐5, where higher scores indicated greater hesitancy [34]. Vaccine confidence was a mean score of a 7-item scale with Likert responses ranging from 1‐5, where higher scores indicated greater confidence [35]. Vaccine-related conspiracy beliefs were a mean score of an adapted 5-item scale with Likert responses ranging from 1‐4, where higher scores indicated greater belief in conspiracy [36]. Vaccine knowledge was a sum score of an adapted 4-item scale with “yes” or “no” responses for each item, where higher scores indicated greater knowledge [37]. Group-based medical mistrust was a sum score of a 12-item scale with Likert responses ranging from 1‐5, where higher scores indicated greater mistrust [38]. For mean scores, if a response was missing for more than one scale item, the mean score was set as missing and not calculated. For summed scores, if a response was missing for any scale item, the summed score was set as missing and not calculated.

### Additional Measures

Once latent profiles were established, differences in several baseline sociodemographic variables and other measures were assessed between the profiles. These included self-reported: sex assigned at birth, gender, age, education (ie, 9th-12th grade; no diploma; high school graduate or GED completed; some college level or technical or vocational degree; bachelor’s degree; other advanced degree), political affiliation (ie, Democrat, Republican, Independent, other), employment status (eg, full-time; part-time; student; not working; disabled, permanently or temporarily), annual income, insurance coverage (eg, public, private, none), social support (continuous scale) [39], and previous flu and human papillomavirus vaccination.

### Outcomes

The primary outcome was uptake of a COVID-19 vaccine anytime during the 3-month follow-up period, based on uploaded vaccine cards or, if not uploaded, self-report at one month and three months. Secondary outcomes included vaccine hesitancy, vaccine confidence, vaccine-related conspiracy beliefs, and vaccine knowledge, which were reported at 3 months. These secondary measures were assessed in the same manner as the baseline latent indicators explained above. Latent profiles were used to assess effect measure modification (EMM) of the TT-C DHI on both primary and secondary outcomes.

### Statistical Analysis

Latent profile analysis allows us to gain insights into “hidden” subgroups in a study sample using person-centered mixture modeling. This approach models how indicator variables relate to each other by estimating different profiles (or classes) and determining the best-fitting model [40,41]. In LPA, model parameters include the probability of an individual belonging to a given profile, and each profile can have its own set of means and variances.

We evaluated two different model specifications: (1) equal variance and covariance across profiles and (2) varied variances and covariances across profiles. For each specification, we tested models with two, three, and four profiles. Model fit was assessed using an analytical hierarchy process based on the following information criteria: Akaike information criterion, Bayesian information criterion, Approximate Weight of Evidence, consistent likelihood criterion, and Kullback information criterion [42]. Lower values indicate a better fit for each criterion. Additional criteria including the sample size-adjusted Bayesian information criterion, entropy, and the bootstrap likelihood ratio (BLRT) were used to assess overall model quality. For these criteria, a lower sample size-adjusted Bayesian information criterion, higher entropy, and statistically significant BLRT indicate a good fit. The best model was selected using an analytical hierarchical process that balanced low values of Akaike information criterion, Bayesian information Criterion, Approximate Weight of Evidence, consistent likelihood criterion, and Kullback information criterion, which indicate a more parsimonious model, with fewer parameters while still accounting for data complexity, with higher entropy to ensure distinct profile separation and a significant BLRT to confirm robustness [42]. After model selection, we assigned study participants to the profile with the highest posterior probability based on their pattern of responses across the variables. Descriptive statistics were then calculated for the sociodemographic and additional variables within each profile. Differences in continuous variables across profiles were tested using ANOVA, while differences in categorical variables were assessed with the *χ*^2^ test of independence.

The primary outcome of vaccine uptake at three months was assessed using a logistic regression model. The odds ratio approximates the risk ratio, as the probability of vaccine uptake was not common (<15%). For secondary outcomes—vaccine hesitancy, vaccine confidence, and vaccine-related conspiracy beliefs—we used linear regression to determine differences in outcomes at three months. Poisson regression was used to test for differences in the secondary outcome of vaccine knowledge score at three months. The main effects of the TT-C DHI intervention on vaccine uptake and vaccine attitudes will be reported in the primary manuscript of the study (Budhwani et al, PhD, unpublished data, 2024). The EMM of the effect of the TT-C DHI intervention on primary and secondary outcomes by profile group was assessed in this study by including the study arm and latent profile at baseline as both main effects and interaction terms. The EMM assesses whether an intervention effect (ie, the effect of the intervention on the outcome in the intervention group compared to the control group) differs across a variable of interest (in this case, the LPA groups). Interaction terms with a P value <.10 were considered indicators of significant effect measure modification. For all models, the state of residence was controlled to account for its inclusion in the stratified randomization study design. We did not control for other variables as they were balanced by the randomized design. All analyses were conducted using R software (version 4.4.1; R Foundation for Statistical Computing) [43].

### Sensitivity Analysis

A sensitivity analysis was conducted using latent class analysis (LCA), typically used with categorical variables, where all indicator variables were dichotomized at appropriate cut-points for each validated scale [44]. We tested LCA models with two, three, and four classes and used similar criteria to assess fit. We then compared the number of classes identified by LCA to the number of profiles identified by LPA, as well as the total variance explained and general sociodemographic differences across the classes/profiles. We chose to report LPA as the main analysis to maintain the continuous nature of the originally validated measures used as indicator variables and because of the added model flexibility of LPA with varying variances and covariances across profiles.

### Ethical Considerations

All study materials and procedures were reviewed and approved by the Institutional Review Boards of the University of North Carolina, Chapel Hill (21‐1746) and Florida State University (STUDY00003617). Informed consent was collected digitally from all study participants before data collection and randomization. All data were deidentified to safeguard participant information.

## Results

A total of 360 Black young adults were included in this study, of whom 76% (n=272) identified as cisgender or transgender women. The median age was 24 (IQR 21‐27) years, and approximately one-third of participants (n=122) had a bachelor’s degree or higher. Overall vaccine uptake at month 3 was low (n=21) and did not significantly differ between the intervention and control arms (odds ratio [OR] 1.88, 95% CI 0.76 to 4.69).

### Latent Profile Analysis

**Figure 1.**
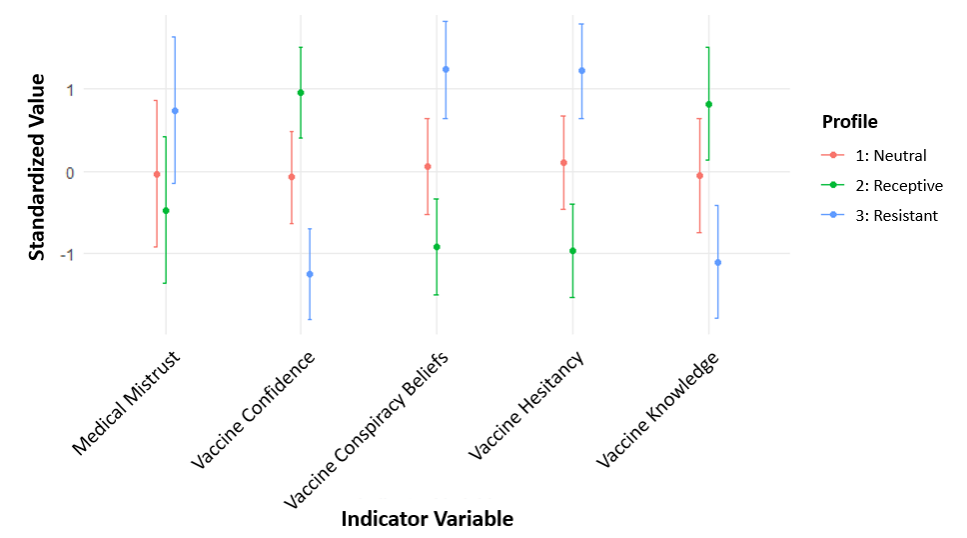
Standardized values of each indicator variable included in the three-profile model that best fit the latent profile analysis of baseline vaccine attitudes in the TT-C study. Points represent the mean standardized value for each profile, with bars showing ± 1 SD. The vaccine-resistant group (Profile 3 in blue) showed the highest medical mistrust, vaccine conspiracy beliefs, and vaccine hesitancy, coupled with the lowest vaccine confidence and knowledge. TT-C: Tough Talks COVID.

Using the prespecified fit criteria with the analytic hierarchy process, we found that the model with three profiles and varied variances and covariances across profiles provided the best fit (Table 1). The profiles in Figure 1 were named based on the mean values of the five indicator variables, all of which were significantly different (*P*<.001) between the profiles (Table 2). The vaccine-receptive group (Profile 2) included 124 participants (34%) and had the lowest mean scores for medical mistrust, conspiracy beliefs, and vaccine hesitancy and the highest mean score for vaccine confidence and knowledge of vaccines compared to other profiles. The vaccine-resistant group (Profile 3) included 81 participants (23%) and had the highest mean score for medical mistrust, conspiracy beliefs, and vaccine hesitancy and the lowest mean score for confidence and knowledge of vaccines compared to other profiles. The vaccine-neutral group (Profile 1) included 155 participants (43%) and had mean scores between those of the vaccine-receptive and vaccine-resistant groups for all indicator variables.

**Table 1.** Fit indices for the latent profile analysis of Model 1 (equal variance and covariance across profiles) and Model 2 (varied variances and covariances across profiles) for 2‐4 profiles of baseline vaccine attitudes in the TT-C^a^ study

Model	Number of profiles	AIC^b^	BIC^c^	AWE^d^	CLC^e^	KIC^f^	SABIC^g^	Entropy	BLRT^h^ *P* value
1	2	4439.42	4501.59	4642.03	4409.15	4458.42	4450.83	0.87	<.001
1	3	4245.05	4330.55	4524.38	4202.71	4270.05	4260.75	0.83	<.001
1	4	4218.36	4327.17	4574.38	4163.96	4249.36	4238.34	0.80	<.001
2	2	3889.83	4049.16	4412.27	3809.06	3933.83	3919.09	0.61	<.001
2	3	3844.34	4035.28	4634.79	3721.77	3909.34	3888.59	0.65	<.001
2	4	3843.89	4180.44	4916.48	3693.39	3943.89	3917.12	0.71	<.001

a TT-C: Tough Talks COVID.

b AIC: Akaike information criterion.

c BIC: Bayesian information criterion.

d AWE: Approximate Weight of Evidence.

e CLC: consistent likelihood criterion.

f KIC: Kullback information criterion.

g SABIC: Sample size-adjusted Bayesian information criterion.

h BLRT: Bootstrap likelihood ratio.

**Table 2.** Sociodemographic and baseline variables of TT-C^a^ study participants by vaccine attitude profile.

Baseline variable	Profile 2 Vaccine-receptive (n=124)	Profile 1 Vaccine-neutral (n=155)	Profile 3 Vaccine-resistant (n=81)	*P* value
Vaccine hesitancy (ranges from 0‐5)				<.001
Median (IQR)	1.8 (1.4-2.0)	2.5 (2.2-2.8)	3.3 (3.0-3.8)	
Mean (SD)	1.7 (0.3)	2.5 (0.5)	3.4 (0.6)	
Vaccination knowledge score (ranges from 0‐4)				<.001
Median (IQR)	4 (3-4)	2 (2-3)	1 (0-1)	
Mean (SD)	3.4 (0.6)	2.2 (0.9)	0.8 (0.8)	
Vaccine confidence (ranges from 0‐5)				<.001
Median (IQR)	4.1 (4.0-4.4)	3.4 (3.1-3.7)	2.6 (2.1-2.9)	
Mean (SD)	4.2 (0.4)	3.4 (0.4)	2.5 (0.5)	
Vaccine conspiracy beliefs (ranges from 0‐5)				<.001
Median (IQR)	1.6 (1.2-1.8)	2.2 (2.0-2.6)	3.1 (3.0-3.6)	
Mean (SD)	1.6 (0.4)	2.3 (0.4)	3.2 (0.5)	
Medical mistrust (ranges from 0‐60)				<.001
Median (IQR)	32 (29-35)	35 (32-38)	39 (36-42)	
Mean (SD)	31.9 (4.6)	34.6 (5.0)	39.4 (6.3)	
Randomization arm, n (%)				.20
Intervention	55 (44)	78 (50)	47 (58)	
Control	69 (56)	77 (50)	34 (42)	
Sex assigned at birth, n (%)				.08
Male	19 (15)	28 (18)	14 (17)	
Female	105 (85)	127 (82)	67 (83)	
Gender identity, n (%)				.70
Man (cis or trans)	17 (14)	25 (16)	15 (19)	
Woman (cis or trans)	93 (75)	119 (77)	60 (74)	
Gender fluid, gender queer, gender nonconforming, nonbinary, agender or other gender	14 (11)	11 (7)	6 (7)	
Age (years)				.05
Median (IQR)	23.0 (20.0-27.0)	24.0 (21.0-26.0)	24.0 (23.0-27.0)	
Mean (SD)	23.3 (3.7)	23.6 (3.3)	24.5 (3.1)	
Hispanic/Latino ethnicity, n (%)	5 (4)	9 (6)	2 (3)	.50
Flu vaccination (ever), n (%)				.05
No	23 (19)	33 (21)	27 (33)	
Yes	97 (78)	114 (74)	48 (59)	
Flu vaccination (past 12 months), n (%)				.01
No	55 (57)	72 (63)	40 (83)	
Yes	40 (41)	33 (29)	7 (15)	
Human papillomavirus (any), n (%)				.12
No	23 (19)	40 (26)	25 (31)	
Yes	83 (67)	86 (55)	39 (48)	
Human papillomavirus vaccination (full series), n (%)				.20
No	8 (9.6)	16 (19)	4 (10)	
Yes	73 (88)	62 (72)	33 (85)	
Political affiliation, n (%)				.001
Democrat	74 (60)	70 (45)	27 (33)	
Republican	2 (1.6)	6 (3.9)	3 (3.7)	
Independent	27 (22)	46 (30)	28 (35)	
Other	8 (6.5)	3 (1.9)	1 (1.2)	
Social support (ranges from 0‐5), n (%)				.02
Median (IQR)	4.2 (3.5-4.8)	3.9 (3.0-4.7)	3.8 (2.6-4.8)	
Mean (SD)	4.1 (0.8)	3.8 (0.9)	3.6 (1.1)	
Annual income (USD), n (%)				.01
≤$24,999	31 (25)	63 (42)	32 (40)	
$25,000 - $49,999	41 (34)	46 (29)	24 (30)	
$50,000 - $74,999	20 (16)	17 (11)	9 (11)	
$75,000 - $99,999	11 (8.9)	11 (7.1)	3 (3.7)	
≥$100,000	12 (9.7)	5 (3.2)	1 (1.2)	
Insurance coverage, n (%)				<.001
None	12 (9.7)	18 (12)	12 (15)	
Private	81 (65)	66 (43)	23 (28)	
Public	27 (22)	59 (38)	40 (49)	
Education level, n (%)				.20
9th to 12th grade, no diploma	8 (6.5)	6 (3.9)	2 (2.5)	
High school graduate or GED completed	22 (18)	42 (27)	24 (30)	
Some college level/technical/vocational degree	45 (36)	50 (32)	36 (44)	
Bachelor’s degree	36 (29)	39 (25)	13 (16)	
Other advanced degree (Master’s, Doctoral degree)	12 (9.7)	17 (11)	5 (6.2)	
Employment status, n (%)				.30
Working, full-time	54 (44)	64 (41)	35 (43)	
Working, part-time	20 (16)	19 (12)	12 (15)	
Not working	14 (11)	26 (17)	11 (14)	
Student	34 (27)	38 (25)	14 (17)	
Disabled, permanently or temporarily	0 (0)	4 (2.6)	3 (3.7)	
Other	2 (1.6)	3 (1.9)	5 (6.2)	

a TT-C: Tough Talks COVID

Individuals in the vaccine-receptive group was significantly more likely to have been vaccinated for the flu in the past 12 months (n=40, 41%) compared with those in the vaccine-neutral (n=33, 29%) and the vaccine-resistant (n=7, 15%) groups (*P*=.01). Additionally, the vaccine-receptive group was more likely to report Democratic political affiliation (n=74, 60%) compared to the vaccine-neutral (n=70, 45%) and vaccinate-resistant (n=27, 33%) groups (*P*=.001). Reported annual income and insurance coverage were highest among the vaccine-receptive group; however, there were no significant differences in educational attainment or employment status across profiles. The vaccine-receptive group also tended to be younger (mean age, 23.3, SD 3.7) years and reported more social support (mean score, 4.1, SD 0.8) compared with both the vaccine-neutral (mean age, 23.6, SD 3.3 years; mean social support score, 3.8, SD, 0.9) and vaccine-resistant (mean age, 24.5, SD 3.1 years; mean social support score, 3.6, SD 1.1) groups (*P*=.05).

### EMM by Baseline Vaccine Attitude Profile

Effect measure modification analysis showed that the TT-C DHI did not have a different effect on vaccine uptake across latent profile subgroups (Table 3). For secondary outcomes, EMM showed that the TT-C DHI had differing effects among those identified as vaccine-resistant compared with those classified as vaccine-receptive at baseline for the outcomes of vaccine hesitancy (interaction *P*=.07), vaccine confidence (interaction *P*=.06), and vaccine-related conspiracy beliefs (interaction *P*=.04) at 3 months (Table 3). There were also differing effects between vaccine-neutral and vaccine-receptive groups from baseline for the outcomes of vaccine hesitancy (interaction *P*=.05) and vaccine confidence (interaction *P*=.05) at 3 months. The vaccine-resistant group showed the greatest effect from the TT-C DHI, with a reduced mean vaccine hesitancy score of −0.40 (95% CI −0.76 to −0.37), an increased mean vaccine confidence score of 0.39 (95% CI 0.02 to 0.75), and a reduced conspiracy belief score of −0.47 (95% CI −0.86 to −0.09). The vaccine-neutral subgroup experienced moderate effects, including a reduced mean vaccine hesitancy score of −0.36 (95% CI −0.52 to −0.19) and an increased mean vaccine confidence score of 0.35 (95% CI 0.18 to 0.51). The TT-C DHI had no significant effect on any of the secondary vaccine-related outcomes among the vaccine-receptive subgroup.

**Table 3.** Results of the effect measure modification assessment for the impact of the TT-C^a^ DHI^b^ on primary and secondary outcomes at month 3 for the three vaccine attitude profiles at baseline.

Outcomes	Odds ratio (95% CI) or Diff (95% CI)^c^	Interaction *P* value^d^
Primary outcomes		
Vaccine uptake (3 months)		.79
Vaccine-resistant	1.81 (0.41-7.90)	
Vaccine-neutral	not enough data	
Vaccine-receptive	1.32 (0.35-5.04)	
Secondary outcomes		
Vaccine hesitancy (3 months)		
Vaccine-resistant	−0.40 (−0.76 to −0.37)	.07
Vaccine-neutral	−0.36 (−0.52 to −0.19)	.05
Vaccine-receptive	−0.08 (−0.26 to 0.09)	–^e^
Vaccine confidence (3 months)		
Vaccine-resistant	0.39 (0.02-0.75)	.06
Vaccine-neutral	0.35 (0.18-0.51)	.05
Vaccine-receptive	0.06 (−0.11 to 0.24)	–
Vaccine knowledge (3 months)		
Vaccine-resistant	0.90 (0.27-1.53)	.12
Vaccine-neutral	0.81 (0.41-1.20)	.17
Vaccine-receptive	0.16 (0.07-0.40)	–
Vaccine-related conspiracy beliefs (3 months)		
Vaccine-resistant	−0.47 (−0.86 to −0.09)	.04
Vaccine-neutral	−0.24 (−0.44 to −0.03)	.36
Vaccine-receptive	−0.09 (−0.26 to 0.08)	–

a TTC: Tough Talks COVID

b DHI: digital health interventions

c Odds ratios (95% CI) are presented for primary outcomes and Diff (95% CI) for secondary outcomes.

d Reference for the interaction *P*-value is the vaccine-receptive subgroup with significance indicated by *P*<.10

e not applicable

### Sensitivity Analysis

When using the prespecified fit criteria with the analytic hierarchy process, we found the LCA model with the best fit to include three classes, similar to the LPA results. Similar to our LPA results, the three classes represented subgroups of study participants who were either: (1) vaccine-receptive, with low mean scores for mistrust, conspiracy beliefs, or hesitancy and high mean scores for knowledge and confidence; (2) vaccine-resistant, with high mean scores for mistrust, conspiracy beliefs, and hesitancy and low mean scores for knowledge or confidence; or (3) somewhere in between the two, as vaccine-neutral. The classes from the LCA had similar differences as the profiles in the LPA analysis with respect to political affiliation, income, insurance coverage, and flu vaccination. Thus, LCA results reinforce the findings from the LPA.

## Discussion

Our LPA of Black young adults participating in an RCT for the TT-C DHI found three distinct subgroups of individuals with different baseline vaccine attitudes. The three subgroups ranged from vaccine-receptive to vaccine-resistant and differed on several sociodemographic variables, such as political affiliation, income, and social support. While there were no significant differences in vaccine uptake by intervention arm between the three subgroups, we identified modification whereby the TT-C DHI had the largest improvement in hesitancy, confidence, and conspiracy beliefs at 3 months among the subgroups who were vaccine-resistant and vaccine-neutral at baseline compared to those who were vaccine-receptive.

To our knowledge, this is the first study to use LPA to explore the efficacy of DHIs for improving vaccine outcomes among Black young adults in the United States South, a population understudied and underserved in vaccine research. Our results from TT-C align with those of several social media and video-based educational DHIs across different populations, which showed the biggest improvements in vaccine outcomes among those with poor vaccine attitudes at baseline [45,46]. Because the TT-C DHI was cocreated with youth from the community and incorporated digital storytelling—a method well suited for marginalized communities [32]—we believe we prevented experiences of “backfiring” or reverse effects seen in more generalized vaccine-related DHIs where vaccine attitudes worsened among those with poor attitudes at baseline [47,48]. Additionally, the vaccine-receptive subgroup in this study had high confidence and knowledge in vaccines, with little hesitancy, mistrust, or conspiracy beliefs despite being insufficiently vaccinated. These baseline scores left limited room for improvement at month 3, which likely explains why TT-C differs from some other vaccine DHIs that saw the largest improvements in vaccine outcomes among those with more positive attitudes at baseline [49,50].

The success of this community-based participatory research project in developing a DHI for Black young adults in the United States South mirrors that of other DHIs aimed at acknowledging and addressing the historical injustices and ongoing inequities that drive distrust within communities of color, while using culturally appropriate messages and existing community infrastructure to address concerns about COVID-19 vaccine safety and access [51-55]. By using digital storytelling to convey accurate, relatable, and culturally resonant messages, DHIs can foster trust and engagement in positive vaccine beliefs, which can serve as a model for improving vaccination among other underserved or vaccine-resistant populations, both for COVID-19 and other vaccines. This work provides a blueprint for public health initiatives to adapt and implement culturally sensitive vaccine interventions that help build the capacity to identify inaccurate or misleading information online and to encourage individuals to make informed decisions. However, it also highlights the challenges of translating changed attitudes into actions.

Despite the significant improvements seen in vaccine attitudes, the lack of impact from the TTC-DHI on actualized vaccination behavior among any subgroup of Black young adults in this study may be attributed to a number of external factors. One key factor was the availability and access of COVID-19 vaccines during the study period of late 2023, when primary series promotion was shifting to boosters. Changing public health guidelines and the short 3-month timeframe in the study may have resulted in a lack of opportunity for behavioral action to get vaccinated, as the recommended frequency of boosters was greater than 3 months and it may have been less likely for clinics to offer the primary vaccination series. Indeed, only 21 (6.4%) participants received a new COVID-19 vaccine over the 3-month follow-up period. Additionally, the Health Belief Model has shown that a change in attitudes is not always enough to effect behavior change; certain conditions, such as perceived risk of infection and resource mobilization, are also required [56,57]. Lastly, other external influences, such as ongoing misinformation and continued structural inequalities in access, may have dampened the direct impact of the intervention on behavior, even as attitudes shifted positively.

This study draws on a large sample of Black young adults from the United States South, and we used a comprehensive range of validated vaccine scales as constructs to conduct the LPA. However, there are several limitations to this work. First, the landscape of COVID-19 science was changing quickly while the app was being developed and implemented (2021‐2024); although app content was responsive to these changes, not all components were adjustable once developed, which may limit the generalizability and overall effectiveness of app messaging. This evolving context may have influenced participants’ attitudes and behaviors in ways that are difficult to fully capture, as new information or policy changes could have overshadowed the effects of the intervention itself. Second, information about vaccine attitudes was collected via self-report, which may be misreported due to social desirability bias. Lastly, the use of LPA does not guarantee proper creation or assignment of subgroups and overlap between identified profiles may impact findings. However, our sensitivity analysis resulted in the same number of subgroups with similar distributions and effect measures, strengthening our confidence in these results and our study conclusions.

This analysis found that Black young adults who are vaccine-resistant or vaccine-neutral may experience larger improvements in vaccine outcomes when using DHIs like TT-C. Future researchers and policymakers can use this knowledge to create better-informed community-based policies, practices, and messaging related to vaccine uptake and attitudes while understanding subpopulations to target for resource efficiency and to drive the biggest improvement in vaccine outcomes. However, vaccine-receptive individuals may experience other benefits from these types of DHIs not assessed, such as improved linkage to vaccine resources or social support and thus should not be excluded. Vaccine-receptive individuals could also be leveraged as peer leaders or vaccine advocates in their respective communities to further increase uptake and acceptability of DHIs among Black young adults in the United States South.

## Abbreviations

BLRT: bootstrap likelihood ratio
DHI: digital health intervention
EMM: effect measure modification
LCA: latent class analysis
LPA: latent profile analysis
TT-C: Tough Talks COVID
